# Long-term endocrine trajectories and reoperation risk after surgery for Rathke’s cleft cysts: a single-center cohort study of 177 patients

**DOI:** 10.1007/s11102-026-01698-2

**Published:** 2026-05-18

**Authors:** Natalia Kremenevski, Waseem Masalha, Daniel Delev, Roland Coras, Arnd Doerfler, Dieter Henrik Heiland, Oliver Schnell

**Affiliations:** 1 Department of Neurosurgery, Universitätsklinikum Erlangen, Friedrich-Alexander University Erlangen-Nürnberg, Schwabachanlage 6, 91054 Erlangen, Germany; 2 Department of Neuropathology, Universitätsklinikum Erlangen, Friedrich-Alexander University Erlangen-Nürnberg, Erlangen, Germany; 3 Department of Neuroradiology, Universitätsklinikum Erlangen, Friedrich-Alexander University Erlangen-Nürnberg, Erlangen, Germany

**Keywords:** Rathke’s cleft cysts, Pituitary endocrine dysfunction, Arginine vasopressin deficiency, MRI volumetry, Chronic cyst-wall inflammation, Reoperation

## Abstract

**Purpose:**

To characterise endocrine trajectories after surgery for Rathke’s cleft cysts (RCCs) and identify factors associated with pituitary hormonal dysfunction and reoperation.

**Methods:**

Retrospective single-centre cohort of 177 patients undergoing RCC surgery (2004–2023). Preoperative MRI volumetry, cyst location, histopathology, and longitudinal endocrine assessments were analysed. Early postoperative MRI defined residual cyst status. Reoperation-free survival was assessed by Kaplan-Meier and multivariable Cox regression.

**Results:**

Patients were 67.8% women (mean age 44.8 ± 17.0 years); median follow-up was 38.5 months. Preoperatively, 50.3% had anterior pituitary dysfunction, 33.3% hyperprolactinaemia, and 9.0% arginine vasopressin deficiency (AVP-D). Larger cyst volume was independently associated with hyperprolactinaemia and gonadal axis dysfunction, whereas age and sex showed axis-specific associations. Cyst location had limited explanatory value. An inflammatory-metaplastic cyst-wall phenotype was associated with more frequent pituitary hormonal dysfunction at presentation. Postoperatively, hyperprolactinaemia resolved in 78.0% of affected patients. AVP-D increased to 24.3% early after surgery and persisted in 22.0% at last follow-up. Gross total resection was achieved in 82.9%, and reoperation occurred in 15.8%. In landmark analysis, residual cyst on early postoperative MRI remained strongly associated with subsequent reoperation after adjustment for age, sex, cyst volume, and location.

**Conclusion:**

Larger cyst volume and an inflammatory-metaplastic cyst-wall phenotype were associated with more frequent pituitary hormonal dysfunction at diagnosis. Most clinically relevant net endocrine changes occurred within the first three postoperative months, supporting standardised reassessment at this time point as a pragmatic early follow-up milestone. Early postoperative MRI residual was strongly associated with reoperation, supporting a function-preserving surgical strategy and risk-adapted follow-up.

**Supplementary Information:**

The online version contains supplementary material available at 10.1007/s11102-026-01698-2.

## Introduction

Rathke’s cleft cysts (RCCs) are benign epithelial cysts of the sellar and suprasellar region and are increasingly identified with modern MRI. While many remain incidental, symptomatic RCCs frequently require surgical treatment due to headache, visual impairment, or anterior and/or posterior pituitary dysfunction potentially related to mass effect and inflammatory involvement of the gland and stalk [1–6]. Although surgery aims to relieve compression and preserve pituitary function, postoperative endocrine outcomes are heterogeneous, and new deficits, particularly disorders of water balance, remain clinically relevant [7–10]. Long-term, axis-specific endocrine trajectories and clinically meaningful timepoints for postoperative endocrine reassessment are not well defined [2, 11].

Beyond endocrine outcomes, radiological recurrence and the need for reoperation represent major long-term challenges. Early postoperative MRI is commonly used to guide surveillance; however, the relationship between radiological residuals, true recurrence after apparent gross total resection, and clinically indicated reoperation remains insufficiently characterised [3, 12, 13]. Surgical strategies often balance symptom relief with pituitary preservation, yet the factors associated with reoperation risk in routine practice remain poorly defined [12, 13].

Emerging data suggest that inflammatory and metaplastic cyst-wall features, including chronic inflammation, squamous metaplasia, xanthogranulomatous change, and proliferative activity, may be associated with pituitary hormonal dysfunction, although their prognostic value beyond anatomical and volumetric parameters remains unclear [3–6, 14]. Whether such histopathological phenotypes are associated with endocrine vulnerability, radiological recurrence, or clinically indicated reoperation remains incompletely understood.

In this retrospective single-centre cohort of surgically treated RCCs, we integrated quantitative MRI volumetry, anatomical location, detailed histopathological phenotyping, and longitudinal endocrine assessment across pituitary axes. We aimed to characterise long-term endocrine trajectories, identify factors associated with pituitary hormonal dysfunction, and assess factors associated with reoperation, with particular emphasis on the prognostic relevance of early postoperative MRI findings. This integrated approach may help refine risk-adapted endocrine follow-up and imaging surveillance after RCC surgery.

## Methods

### Patient population

This retrospective, single-centre cohort study included all adult patients (≥ 18 years) with histologically confirmed RCCs who underwent surgery at the Department of Neurosurgery, Universitätsklinikum Erlangen, Friedrich-Alexander University Erlangen-Nuremberg between January 2004 and July 2023. The study was approved by the institutional ethics committee (No.25-164-Br) and complied with the Declaration of Helsinki (revision 2013).

### Data collection

Demographic and baseline clinical characteristics, presenting symptoms, pre- and postoperative endocrine profiles, imaging findings, surgical approach, histopathological results, perioperative complications and follow-up data were retrospectively extracted from electronic medical records. Age at diagnosis was the age at first operation. For symptomatic patients, the primary symptom was the leading patient-reported reason for seeking medical attention (exclusive, one per patient). Secondary symptoms captured any additional complaints present at presentation (non-exclusive). Incidental RCCs were asymptomatic lesions detected on unrelated imaging.

### Assessment of pituitary function

Endocrine evaluation followed established criteria [15] and included serum cortisol, thyroid-stimulating hormone (TSH), free thyroxine (fT4), follicle-stimulating hormone (FSH), luteinising hormone (LH), testosterone (men), estradiol (women), growth hormone (GH), insulin-like growth factor 1 (IGF-1), prolactin, and plasma adrenocorticotropic hormone (ACTH). Endocrine abnormalities were defined as follows: secondary adrenal insufficiency (SAI), basal cortisol < 5 µg/dL and/or peak < 18 µg/dL after a 250-µg Synacthen test; thyrotropic dysfunction, low fT4 with low/inappropriately normal TSH, or levothyroxine substitution, as the indication for substitution could not be reliably determined as central versus primary thyroid disease in this retrospective cohort; hyperprolactinaemia, prolactin above the laboratory reference range; gonadal axis dysfunction: in men, low testosterone with low/inappropriately normal gonadotrophins; in premenopausal women, low oestradiol or compatible symptoms (e.g. oligomenorrhoea, amenorrhoea, infertility) with low/inappropriately normal gonadotrophins; in postmenopausal women, suppressed LH/FSH with low oestradiol; IGF-1-defined somatotropic impairment, age-/sex-adjusted low IGF-1 (no GH stimulation testing given the retrospective design); arginine vasopressin deficiency (AVP-D), polyuria with hypotonic urine and/or hypernatraemia, clinical documentation of AVP-D, or desmopressin requirement; persistent AVP-D at follow-up, ongoing desmopressin requirement and/or documented persistent polyuria-polydipsia, as standardised desmopressin withdrawal testing was not routinely performed. Pituitary status was graded as partial hypopituitarism (≥ 1 but not all anterior axes impaired) or complete hypopituitarism (all anterior axes impaired).

### Imaging evaluation

Preoperative pituitary MRI assessed cyst size and anatomical location. Maximal diameters were measured in three orthogonal planes - anteroposterior (A), mediolateral (B), craniocaudal (C) - and recorded in millimetres. Volume was estimated by a modified ellipsoid formula (A×B×C/2). By extension, RCCs were categorised as intrasellar, intrasellar with suprasellar extension, or purely suprasellar.

### Surgical approach

Surgery was undertaken for symptomatic RCCs, including headache, visual impairment or endocrine/water-balance dysfunction, and/or relevant radiological progression, as documented in the clinical records. The standard approach was transsphenoidal microsurgery; transcranial access was reserved for selected anatomical scenarios. Surgery primarily consisted of cyst fenestration/evacuation with selective removal of accessible cyst-wall tissue for histopathology. Gland/stalk preservation was prioritised, and extensive cyst-wall resection was not routinely pursued. Extent of resection was assessed on the first early postoperative MRI, usually obtained at approximately 3 months.

### Neuropathological examinations

Surgical specimens were stained with H&E and PAS; immunohistochemistry included Ki-67 (MIB-1) and cytokeratins (e.g. AE1/AE3, CK5, CK7). Pathology reports recorded epithelial phenotype, squamous metaplasia, cyst-wall and dural inflammation, xanthogranulomatous reaction, hypophysitis, and the Ki-67 labeling index.

### Follow-up and recurrence

Preoperatively, all patients underwent standard neurological, ophthalmological and endocrinological assessment. Follow-up was scheduled on postoperative day 7 (POD7), at 3 months, and annually. Baseline and POD7 analyses used the full cohort (*n* = 177), 3-month analyses included 170 patients with available paired data; last-follow-up analyses were restricted to the single-operation subset. Pituitary MRI was obtained at approximately 3 months and then annually. Gross total resection (GTR) was defined as no residual cyst on the 3-month MRI; subtotal resection (STR) as any visible remnant. Recurrence was reappearance after documented GTR or growth of a residual by ≥ 3 mm on any MRI beyond the 3-month scan. Reoperation was reserved for clinically indicated symptomatic recurrence.

### Statistical analysis

Analyses were performed in IBM SPSS Statistics (v29). Continuous variables are reported as mean ± SD or median (IQR), according to distribution assessed by Shapiro-Wilk testing; variance homogeneity was assessed using Levene’s test. Between-group comparisons used independent-samples t test or Mann-Whitney U tests; for > 2 groups, Kruskal-Wallis test. Categorical variables were compared using χ² or two-sided Fisher’s exact tests. Paired changes were analysed using paired t tests or Wilcoxon signed-rank tests for continuous variables, McNemar’s exact test for categorical variables, and Cochran’s Q test for > 2 time points. Reoperation-free survival was estimated using Kaplan-Meier analysis with Greenwood standard errors; unadjusted comparisons by postoperative residual cyst status used log-rank testing. Cox regression analysed time from the first early postoperative MRI to reoperation or last follow-up. Because residual cyst status was defined on this MRI, a landmark approach was used. In one patient reoperated before the scheduled 3-month MRI, a symptom-triggered MRI at 2.1 months defined residual status. The primary Cox model adjusted for age, sex, cyst volume, and cyst location; histopathological variables were evaluated only in separate exploratory extended models to avoid overfitting. Results are reported as hazard ratios (HRs), 95% confidence intervals (CIs), and two-sided p values. Multivariable logistic regression models were fitted separately for each preoperative endocrine outcome, including cyst volume per 1 cm³, age per year, sex with female as reference, and cyst location with intrasellar as reference. Results are reported as adjusted odds ratios (aORs), 95% CIs, and two-sided p values. Complete-case analysis was used. Women using oral contraceptives (*n* = 11) were excluded from the gonadal axis model. For Table 4, Benjamini-Hochberg false discovery rate (FDR) correction was applied across all univariable histopathology-axis tests; adjusted p values are reported as q values. Two-sided α = 0.05 unless stated otherwise.

## Results

### Patient characteristics and clinical presentation

#### Baseline characteristics

The cohort included 177 patients, with a female predominance (120 women, 67.8%; female-to-male ratio 2.1:1). Mean age at surgery was 44.8 ± 17.0 years overall; women were younger than men (42.0 ± 16.1 vs. 50.9 ± 17.2 years; *p* < 0.001). Most patients (84.2%) underwent a single procedure, whereas 28 (15.8%) required reoperation for symptomatic recurrence/progression. Median follow-up was 38.5 months (IQR 11.8–89.0; range 3-248) (Table 1).

**Table 1:** Baseline characteristics and key outcomes

Characteristic	Value
Cohort size, n	177
Sex, n (%)	
- women	120 (67.8)
- men	57 (32.2)
Age at surgery, years, mean ± SD	44.8 ± 17.0
- women	42.0 ± 16.1 (*p* < 0.001‡)
- men	50.9 ± 17.2
Follow-up, months, median (IQR; range)	38.5 (11.8–89.0; 3-248)
BMI, kg/m², mean ± SD	
- paired pre-op → 3 months (*n* = 125)	26.02 ± 5.12 → 26.62 ± 5.18 (*p* = 0.002†)
- paired pre-op → last FU (single-op cohort, *n* = 68)	25.32 ± 4.96 → 27.12 ± 5.31 (*p* < 0.001†)
- paired 3 months → last FU (single-op cohort, *n* = 63)	25.90 ± 4.87 → 26.91 ± 5.21 (*p* < 0.001†)
Cyst location, n (%)	
- intrasellar	32 (18.1)
- suprasellar	29 (16.4)
- intra-/suprasellar	116 (65.5)
Surgical approach, n (%)	
- transsphenoidal	167 (94.4)
- transcranial	10 (5.6)
Early postoperative MRI available (~ 3 months), n/N (%)	170/177 (96.0)
Extent of resection on early MRI, n/N (%)	
- GTR	141/170 (82.9)
- STR	29/170 (17.1)
Reoperation required, n (%)	28 (15.8)
- women	25/120 (20.8) (*p* = 0.008§)
- men	3/57 (5.3)
Time to first reoperation, months, median (IQR; range)	45.7 (20.0-76.1; 2.1-168.4)
Reoperation rate: STR vs. GTR, n/N (%)	12/29 (41.4) vs. 16/141 (11.3) (*p* < 0.001§)
Reoperation-free survival, % at 1 / 3 / 5 / 10 years	97.1 / 89.1 / 82.2 / 67.9

Notes. Values are n (%) unless stated otherwise. Continuous variables are reported as mean ± SD or median (IQR; range), as indicated. Early postoperative MRI refers to imaging obtained at approximately 3 months after surgery; extent of resection (GTR/STR) was defined on early postoperative MRI. Long-term BMI analyses were restricted to patients undergoing a single operation with paired measurements available. †Paired t test (two-sided). ‡Between-sex comparison (two-sided). §Between-group comparison (two-sided). Abbreviations: BMI, body mass index; FU, follow-up; GTR, gross total resection; STR, subtotal resection; MRI, magnetic resonance imaging

RCCs most often involved both intrasellar and suprasellar regions (65.5%), whereas isolated intrasellar and suprasellar locations were less common (18.1% and 16.4%). Intra-/suprasellar cysts were larger than intrasellar or suprasellar lesions (1.72 ± 1.55 vs. 0.66 ± 0.52 and 0.65 ± 0.68 cm³; both *p* < 0.001).

#### Clinical presentation

Headache was the most frequent leading presenting symptom (*n* = 61, 34.5%) and the most frequent manifestation overall (*n* = 89, 50.3%). Visual impairment was the second most common leading symptom (*n* = 18, 10.2%) and represented the main mass-effect manifestation, occurring overall in 36 patients (20.4%). Gonadal dysfunction and fatigue were common additional manifestations, occurring overall in 61 (34.5%) and 53 (29.9%) patients, respectively. In 48 patients (27.1%), the RCC was an incidental finding (Table 2).

**Table 2:** Clinical presentation at diagnosis

Clinical manifestation	Leading presenting symptom, *n* (%)	Present overall, *n* (%)
Headache	61 (34.5%)	89 (50.3%)
Incidental finding	-	48 (27.1%)
Visual impairment	18 (10.2%)	36 (20.4%)
Gonadal dysfunction^*^	16 (9.0%)	61 (34.5%)
AVP-D	13 (7.3%)	16 (9.0%)
Fatigue	9 (5.1%)	53 (29.9%)
Hyponatremia	6 (3.4%)	6 (3.4%)
Galactorrhea	4 (2.3%)	13 (7.4%)
Weight gain	2 (1.1%)	17 (9.6%)
Hirsutism	-	1 (0.6%)
CN III palsy	-	1 (0.6%)

Notes. Values are n (%) of the full cohort (*n* = 177). Leading presenting symptom denotes the main patient-reported reason for presentation and was mutually exclusive. Present overall includes leading and additional manifestations and was therefore not mutually exclusive, except for incidental findings, which denote asymptomatic RCCs detected on imaging for unrelated indications. Abbreviations: AVP-D, arginine vasopressin deficiency; CN III, oculomotor nerve. * Includes menstrual irregularities, amenorrhoea, reduced libido, and erectile dysfunction

Leading presenting symptom was associated with cyst volume (*p* = 0.008), with the largest volumes in patients presenting with visual impairment (median 2.30 cm³, IQR 1.04–3.50) and smaller volumes in those presenting with headache (median 0.92 cm³, IQR 0.51–1.69). Endocrine and water‑balance presentations were also associated with cyst‑wall inflammation (*p* < 0.001), which was most frequent in patients presenting with gonadal dysfunction or AVP‑D and less common in incidental cases. Compared with other presentations, headache showed a higher prevalence of cyst‑wall inflammation (61.2% vs. 38.8%; *p* = 0.001).

### Endocrine function.

#### Preoperative endocrine function

At diagnosis, 88 patients (49.7%) had normal anterior pituitary function, 73 (41.2%) partial and 16 (9,0%) complete hypopituitarism, resulting in an overall prevalence of endocrine dysfunction of 50.3%. By axis, gonadal axis dysfunction was present in 67 patients (37.9%), with additional 11 women (6.2%) using oral contraceptives (excluded from gonadal-axis analyses); SAI in 39 (22.0%); hyperprolactinaemia in 59 (33.3%); IGF-1-defined somatotropic impairment in 34 (19.2%); thyrotropic dysfunction in 61 (34.5%), comprising low fT4 in 13 patients (7.3%) and levothyroxine substitution in 48 (27.1%); and AVP-D in 16 (9.0%).

In multivariable logistic regression models adjusted for age, sex, and cyst location (reference category: intrasellar; sex reference: female), cyst volume was independently associated with hyperprolactinaemia (aOR 1.48 per 1 cm³, 95% CI 1.12–1.96; *p* = 0.006) and gonadal axis dysfunction (aOR 1.35 per 1 cm³, 95% CI 1.03–1.76; *p* = 0.028), whereas the association with SAI did not reach statistical significance (aOR 1.28 per 1 cm³, 95% CI 0.99–1.66; *p* = 0.062) (Table 3). Age was independently associated with SAI (aOR 1.03 per year, 95% CI 1.00-1.05; *p* = 0.026) and thyrotropic dysfunction (aOR 1.04 per year, 95% CI 1.02–1.07; *p* < 0.001). Male sex was associated with higher odds of SAI (aOR 2.42, 95% CI 1.10–5.35; *p* = 0.029) and gonadal axis dysfunction (aOR 2.93, 95% CI 1.43-6.00; *p* = 0.003), and with lower odds of hyperprolactinaemia (aOR 0.43, 95% CI 0.19–0.95; *p* = 0.037). Suprasellar location was associated with lower odds of thyrotropic dysfunction (aOR 0.25, 95% CI 0.07–0.83; *p* = 0.024), whereas cyst location was not independently associated with the other endocrine outcomes (all *p* > 0.05). Preoperative AVP-D was not independently associated with cyst volume, age, sex, or cyst location (all *p* > 0.05).

**Table 3:** Multivariable logistic regression analyses of preoperative endocrine dysfunction

Outcome	Events / *N*	Covariate	aOR (95% CI)	*p* value
Hyperprolactinaemia	59 / 177	Cyst volume (per 1 cm³)	1.48 (1.12–1.96)	0.006
		Age (per year)	0.99 (0.97–1.01)	0.512
		Male sex	0.43 (0.19–0.95)	0.037
		Suprasellar vs. intrasellar	0.52 (0.15–1.85)	0.311
		Intra-/suprasellar vs. intrasellar	1.03 (0.40–2.64)	0.954
SAI	39 / 177	Cyst volume (per 1 cm³)	1.28 (0.99–1.66)	0.062
		Age (per year)	1.03 (1.00-1.05)	0.026
		Male sex	2.42 (1.10–5.35)	0.029
		Suprasellar vs. intrasellar	0.38 (0.09–1.55)	0.178
		Intra-/suprasellar vs. intrasellar	0.62 (0.22–1.76)	0.368
IGF-1-defined somatotropic impairment	34 / 177	Cyst volume (per 1 cm³)	1.23 (0.95–1.59)	0.123
		Age (per year)	1.01 (0.98–1.03)	0.570
		Male sex	2.09 (0.93–4.70)	0.074
		Suprasellar vs. intrasellar	1.12 (0.31–4.09)	0.869
		Intra-/suprasellar vs. intrasellar	0.84 (0.28–2.51)	0.755
Gonadal axis dysfunction	67 / 166	Cyst volume (per 1 cm³)	1.35 (1.03–1.76)	0.028
		Age (per year)	0.99 (0.97–1.01)	0.184
		Male sex	2.93 (1.43-6.00)	0.003
		Suprasellar vs. intrasellar	0.40 (0.13–1.30)	0.129
		Intra-/suprasellar vs. intrasellar	0.72 (0.29–1.79)	0.480
Thyrotropic dysfunction	61 / 177	Cyst volume (per 1 cm³)	1.03 (0.81–1.31)	0.810
		Age (per year)	1.04 (1.02–1.07)	< 0.001
		Male sex	0.61 (0.29–1.30)	0.200
		Suprasellar vs. intrasellar	0.25 (0.07–0.83)	0.024
		Intra-/suprasellar vs. intrasellar	0.52 (0.21–1.30)	0.160
AVP-D	16 / 177	Cyst volume (per 1 cm³)	0.67 (0.34–1.30)	0.232
		Age (per year)	1.02 (0.99–1.06)	0.181
		Male sex	0.74 (0.23–2.41)	0.619
		Suprasellar vs. intrasellar	0.31 (0.05–1.85)	0.200
		Intra-/suprasellar vs. intrasellar	0.49 (0.13–1.83)	0.289

Notes. Adjusted odds ratios (aORs) with 95% confidence intervals (CIs) are shown. Models were adjusted for age, sex, cyst volume, and cyst location. Female sex and intrasellar location served as reference categories. Women using oral contraceptives (*n* = 11) were excluded from the gonadal axis model. Thyrotropic dysfunction comprised low fT4 and/or levothyroxine substitution. IGF-1-defined somatotropic impairment was defined by age- and sex-adjusted low IGF-1. AVP-D, arginine vasopressin deficiency; SAI, secondary adrenal insufficiency

Several cyst-wall histopathological features were associated with preoperative endocrine dysfunction in exploratory univariable analyses (Table 4). Benjamini-Hochberg FDR correction was applied across all histopathology-axis combinations, with FDR-adjusted p values reported as q values. After FDR correction, hyperprolactinaemia, gonadal axis dysfunction, SAI, and AVP-D each showed significant associations with selected inflammatory, metaplastic, or proliferative features (all q < 0.05), whereas no significant correlates were identified for IGF-1-defined somatotropic impairment or thyrotropic dysfunction. These analyses were unadjusted; multivariable models assessing cyst volume, anatomical location, age, and sex for each endocrine outcome are reported in Table 3.

**Table 4:** Exploratory univariable associations between histopathological features and preoperative pituitary hormonal dysfunction by axis

Histopathology	Prolactin ↑ (q)	Adrenal insufficiency (q)	IGF-1-defined somatotropic impairment (q)	Gonadal axis dysfunction (q)	Thyrotropic dysfunction (q)	AVP-D (q)
Ki-67 ≥ 3%	0.009	0.039	0.875	0.006	0.142	0.045†
Squamous metaplasia	0.005	0.077	0.241	0.010	0.496	0.196†
Chronic cyst-wall inflammation	0.004	0.202	0.343	0.003	0.620	0.029†
Chronic dural inflammation	0.072	0.020	0.569	0.003	0.853	0.279†
Xanthogranulomatous inflammation	0.018†	1.000†	0.975†	0.440†	0.691†	0.140†
Hypophysitis	0.072†	0.140†	0.956†	0.174†	0.232†	0.360†

Notes. Entries are q values from exploratory univariable, unadjusted analyses (Benjamini-Hochberg FDR-adjusted p values) computed across all histopathology-axis combinations in this table; q < 0.05 indicates associations remaining significant after correction for multiple testing. Pearson’s χ² test (two-sided) was used unless expected cell counts were < 5, in which case Fisher’s exact test was applied (†). For AVP-D (*n* = 16), Fisher’s exact test was used for all comparisons. Women using oral contraceptives (*n* = 11) were excluded from gonadal-axis analyses. For the thyrotropic axis, dysfunction was defined as low/substituted fT4 (low fT4 and/or levothyroxine substitution). Abbreviations: AVP-D, arginine vasopressin deficiency; Ki-67, proliferation index

#### Early postoperative endocrine function

**Fig. 1:**
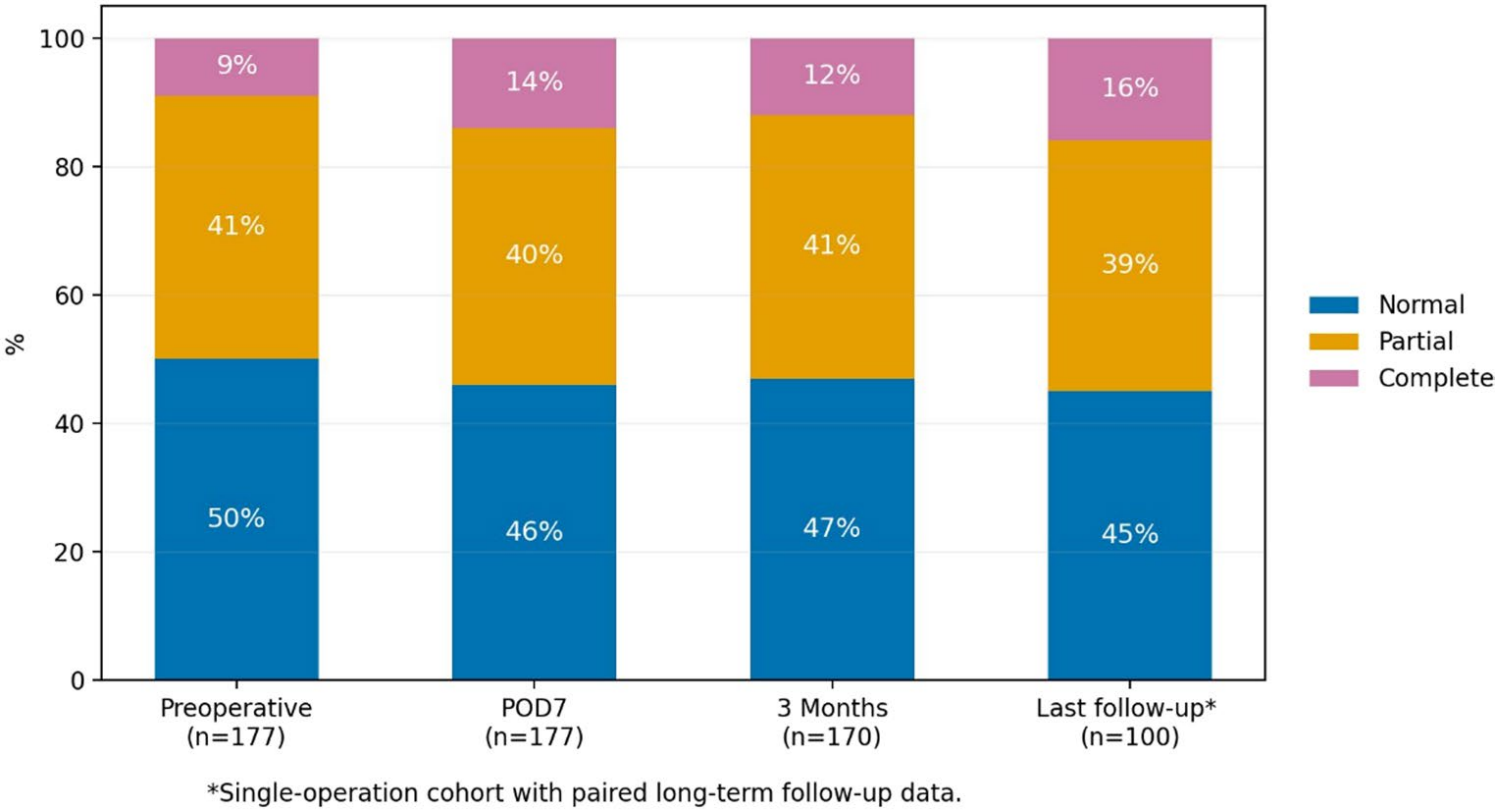
Global anterior pituitary function over time. Distribution of global anterior pituitary status, classified as normal function, partial hypopituitarism, or complete hypopituitarism, preoperatively (*n* = 177), at postoperative day 7 (POD7; *n* = 177), at 3 months (*n* = 170), and at last follow-up (*n* = 100). Values are percentages. Last follow-up is restricted to the single-operation cohort with paired 3-month and long-term endocrine follow-up

Global anterior pituitary status at POD7 differed significantly from the preoperative distribution in the full cohort (*n* = 177; *p* < 0.001) (Fig. 1). Overall, 43 patients (24.3%) showed new or worsened hormonal dysfunction, whereas 134 (75.7%) were stable or improved. Axis-specific paired changes are detailed in Table S1. Hyperprolactinaemia resolved in 78.0% of affected patients and newly developed in 4.2% (*p* < 0.001). The prevalence of SAI increased from 22.0% to 27.1%; 11.6% developed new SAI and 17.9% improved, with no significant net change (*p* = 0.093). New SAI was associated with larger cyst volume (*p* = 0.043), whereas no association with location was observed. New gonadal axis dysfunction occurred in 16.2% and was unrelated to cyst volume or location. Descriptively, SAI and gonadal axis dysfunction were more frequent after transcranial surgery, but this should be interpreted cautiously given the small subgroup (*n* = 10; SAI 40.0% vs. 7.2%; gonadal axis dysfunction 37.5% vs. 8.2%). Thyrotropic dysfunction increased from 34.5% to 44.6% (*p* < 0.001) without clear anatomical or surgical correlates. IGF-1 alterations occurred in 18.6%: 8.4% developed new IGF-1-defined somatotropic impairment, while 38.2% of those with preoperative impairment normalised; overall, somatotropic status remained unchanged and was unrelated to cyst size, location, or approach. AVP-D was present preoperatively in 16 patients (9.0%) and persisted in 15. Among those without preoperative AVP-D, 28 (17.4%) developed new-onset AVP-D, yielding a postoperative prevalence of 24.3% (*p* < 0.001). New AVP-D was not associated with cyst volume or cyst location, and no clear descriptive trend was observed. Descriptively, new AVP-D was more frequent after transcranial than transsphenoidal surgery (50.0% vs. 13.8%), although this comparison should be interpreted cautiously given the small transcranial subgroup.

#### Endocrine function at 3 months after surgery

At 3 months, global anterior pituitary status is shown in Fig. 1 (*n* = 170). Relative to POD7, 88.8% (71/80) of patients with initially normal anterior pituitary function remained normal and 11.3% (9/80) developed new partial insufficiency; none progressed to complete. Among patients with partial insufficiency, 13.4% (9/67) normalised, 85.1% (57/67) remained partial, and 1.5% (1/67) worsened to complete. Of those with complete insufficiency at POD7, 87.0% (20/23) remained complete and 13.0% (3/23) improved to partial. Overall redistribution was significant (*p* < 0.001). Across individual axes, corticotropic function showed no relevant net change, and POD7 prolactin normalisation was maintained. IGF-1-defined somatotropic status showed modest net deterioration (*p* = 0.008), driven by new low IGF-1 in a subset of patients with normal POD7 values. Thyrotropic status showed no net change; most early abnormalities transitioned to substitution, and interpretation remains limited because levothyroxine could not always be attributed to central versus primary thyroid disease. For the gonadal axis, the three-category distribution (normal/low/on contraceptives) was conserved, whereas binary analysis excluding contraceptive users showed net improvement (*p* = 0.008). AVP-D remained highly persistent, with unchanged prevalence from POD7 to 3 months (24.0%); 97.6% (40/41) of early AVP-D cases persisted, and only one new case occurred (Table S2).

#### Endocrine function at last follow-up

Long-term endocrine trajectory analyses were restricted to the single-operation cohort (*n* = 149), of whom 100 had paired endocrine data at both 3 months and last follow-up, whereas 49 did not. Cohort selection is shown in Figure S1, and baseline characteristics of patients with versus without paired 3-month/long-term follow-up are summarised in Table S3. In the paired subgroup (*n* = 100), last follow-up anterior pituitary status was normal in 45.0%, partial in 39.0% and complete in 16.0% (Fig. 1). Compared with the 3-month assessment, 90.9% of patients with normal anterior pituitary function remained normal and 9.1% deteriorated to partial insufficiency; none progressed to complete insufficiency. Of those with partial insufficiency at 3 months, 11.9% improved to normal, 83.3% remained partial and 4.8% worsened to complete insufficiency. All patients with complete insufficiency at 3 months remained complete at last follow-up. Overall redistribution of anterior pituitary function between 3 months and last follow-up was significant (*p* < 0.001). In axis-specific paired analyses, no significant net change was observed between 3 months and last follow-up in any anterior pituitary axis or in AVP-D.

#### Endocrine function over time

Global anterior pituitary status changed significantly over time (Fig. 1). It shifted from preoperative 49.7/41.2/9.0% (normal/partial/complete) to 46.3/39.5/14.1% at POD7 (*p* < 0.001 vs. preoperative). At 3 months, marginal proportions were similar (47.1/40.6/12.4%); however, redistribution from POD7 to 3 months was significant in paired data (*n* = 170; *p* < 0.001). In the paired single-operation cohort (*n* = 100), last-follow-up proportions were 45.0/39.0/16.0%, with significant redistribution from 3 months (*p* < 0.001). Last-follow-up estimates are based on the paired single-operation cohort (*n* = 100) and are not directly comparable with baseline (*n* = 177).

**Fig. 2:**
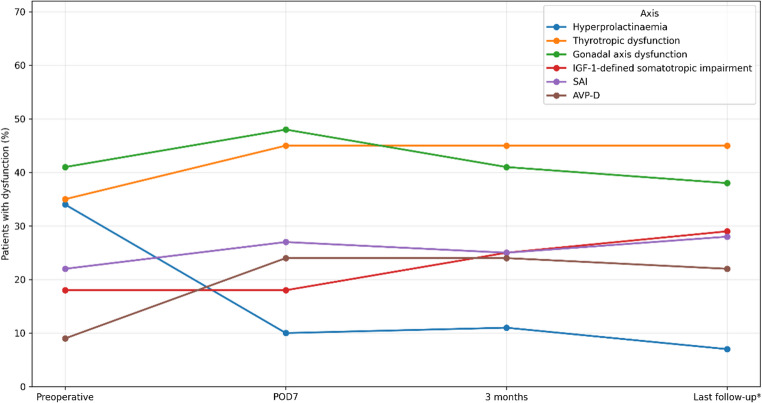
Axis-specific endocrine dysfunction trajectories over time. Lines show the percentage of patients with axis-specific dysfunction preoperatively (*n* = 177), at POD7 (*n* = 177), at 3 months (*n* = 170), and at last follow-up (*n* = 100). *Last follow-up is restricted to the paired single-operation cohort. Gonadal-axis analyses exclude women using oral contraceptives. Thyrotropic dysfunction was defined as low/substituted fT4 (low fT4 and/or levothyroxine substitution), and somatotropic impairment as low age-/sex-adjusted IGF-1 without GH stimulation testing. Abbreviations: AVP-D, arginine vasopressin deficiency; POD7, postoperative day 7; SAI, secondary adrenal insufficiency

Axis-specific trajectories are summarised in Table 5; Fig. 2. Prolactin improved promptly after surgery (*p* < 0.001), with no further significant change. Thyrotropic dysfunction increased by POD7 and remained higher than baseline thereafter (*p* ≤ 0.002), although interpretation is limited because central hypothyroidism could not always be distinguished from primary thyroid disease in this retrospective cohort. IGF-1-defined somatotropic impairment showed no net change at POD7 but increased by 3 months (preoperative vs. 3 months *p* < 0.001; POD7 vs. 3 months *p* = 0.008), and remained evident at last follow-up (preoperative vs. last follow-up *p* = 0.015). Corticotropic function showed no significant net change. The gonadal axis, excluding contraceptive users, demonstrated early recovery from POD7 to 3 months (*p* = 0.008) and was otherwise stable. AVP-D increased from preoperative assessment to POD7 (*p* < 0.001), remained unchanged at 3 months, and remained higher than baseline at last follow-up (*p* < 0.001).

**Table 5:** Endocrine dysfunction over time and paired within-patient comparisons by pituitary axis

Axis	Preoperative dysfunction, % (*n* = 177)	POD7 dysfunction, % (*n* = 177)	3-month dysfunction, % (*n* = 170)	Last FU dysfunction, % (*n* = 100)†	p Pre→POD7 (paired *n* = 177)	p Pre→3 mo (paired *n* = 170)	p Pre→Last FU† (paired *n* = 100)	p POD7→3 mo (paired *n* = 170)	p POD7→Last FU† (paired *n* = 100)	p 3 mo→Last FU† (paired *n* = 100)
Hyperprolactinaemia	34	10	11	7	< 0.001	< 0.001	< 0.001	0.629	0.359	0.332
Thyrotropic dysfunction	35	45	45	45	< 0.001	0.002	0.001	0.581	0.189	0.581
Gonadal axis dysfunction	41	48	41	38	0.012	< 0.001	1.000	0.008	0.189	0.774
IGF-1-defined somatotropic impairment	18	18	25	29	1.000	< 0.001	0.015	0.008	0.001	0.210
SAI	22	27	25	28	0.093	0.210	0.124	0.754	0.629	0.508
AVP-D	9	24	24	22	< 0.001	< 0.001	< 0.001	1.000	0.388	0.388

Notes. Values are percentages of patients with axis-specific dysfunction at each time point. Preoperative and POD7 analyses include the full cohort (*n* = 177); the 3-month assessment includes patients with available follow-up (*n* = 170). Last follow-up is restricted to the paired single-operation cohort (*n* = 100)†. Paired p values reflect within-patient changes between the indicated time points and were calculated using two-sided McNemar’s test on paired data, with sample sizes shown in the column headers. For gonadal-axis analyses, denominators differ because women using oral contraceptives were excluded. For the thyrotropic axis, dysfunction was defined as low fT4 and/or levothyroxine substitution. Abbreviations: SAI, secondary adrenal insufficiency; AVP-D, arginine vasopressin deficiency; POD7, postoperative day 7; FU, follow-up

### Body mass index over time

BMI increased from 26.02 ± 5.12 to 26.62 ± 5.18 kg/m² at 3 months (*n* = 125; +0.60 kg/m²; *p* = 0.002) and from 25.32 ± 4.96 to 27.12 ± 5.31 kg/m² at last follow-up in the single-operation cohort (*n* = 68; +1.81 kg/m²; *p* < 0.001) (Table 1).

### Surgical complications

SIADH was the most common complication (41, 23.2%), followed by meningitis (14, 7.9%) and CSF leak (9, 5.1%). Other complications each occurred in ≤ 4.0% and are detailed in Table S4. The distribution of complication types was not associated with cyst location (*p* = 0.377). In the small transcranial subgroup (*n* = 10), acute visual deterioration was more frequent than after transsphenoidal surgery (20.0% vs. 0.6%); postoperative haemorrhage and chronic subdural haematoma occurred only in this subgroup. These findings are descriptive.

### MRI-defined extent of resection

Early postoperative MRI was available in 170/177 patients (96.0%). Radiological GTR was achieved in 82.9% and STR in 17.1% of patients with available early MRI (Table 1). STR frequency did not differ by cyst location (*p* = 0.832) or cyst volume (*p* = 0.341).

### Reoperation incidence, associated factors, and reoperation-free survival

Reoperation was performed in 28/177 patients (15.8%) after a median of 45.7 months (IQR 20.0-76.1; range 2.1-168.4). Early postoperative MRI residual was strongly associated with subsequent reoperation (12/29 [41.4%] after STR vs. 16/141 [11.3%] after GTR; *p* < 0.001). Notably, 16/28 (57.1%) reoperations occurred despite MRI-defined GTR, consistent with later radiological recurrence, whereas 17/29 (58.6%) early residuals were managed conservatively. Female sex was associated with a higher reoperation risk in unadjusted analysis (20.8% vs. 5.3%; *p* = 0.008), not explained by differences in early residual prevalence (18.8% vs. 13.2%; *p* = 0.37).

Of the 170 patients with early postoperative MRI, 167 had valid follow-up and were therefore included in the landmark time-to-event analyses, including 139 without residual cyst and 28 with residual cyst on the first early postoperative MRI. In multivariable Cox regression adjusted for age at surgery, sex, cyst volume, and cyst location, residual cyst on early postoperative MRI remained significantly associated with reoperation risk (male vs. female: HR 4.06, 95% CI 1.84–8.97; *p* < 0.001). By contrast, sex was no longer independently associated with reoperation after adjustment (HR 0.36, 95% CI 0.11–1.26; *p* = 0.109). Age at surgery, cyst volume, and cyst location were likewise not significantly associated with reoperation.

In additional exploratory analyses, histopathological variables were evaluated in separate extended Cox models to avoid overfitting. The association between early postoperative residual cyst and reoperation remained robust after additional adjustment for Ki-67 and squamous metaplasia (HR 4.69, 95% CI 1.93–11.39; *p* < 0.001), chronic cyst-wall inflammation and xanthogranulomatous inflammation (HR 4.24, 95% CI 1.82–9.92; *p* < 0.001), and hypophysitis and chronic dural inflammation (HR 3.67, 95% CI 1.53–8.76; *p* = 0.003). None of the tested histopathological variables was independently associated with reoperation.

**Fig. 3:**
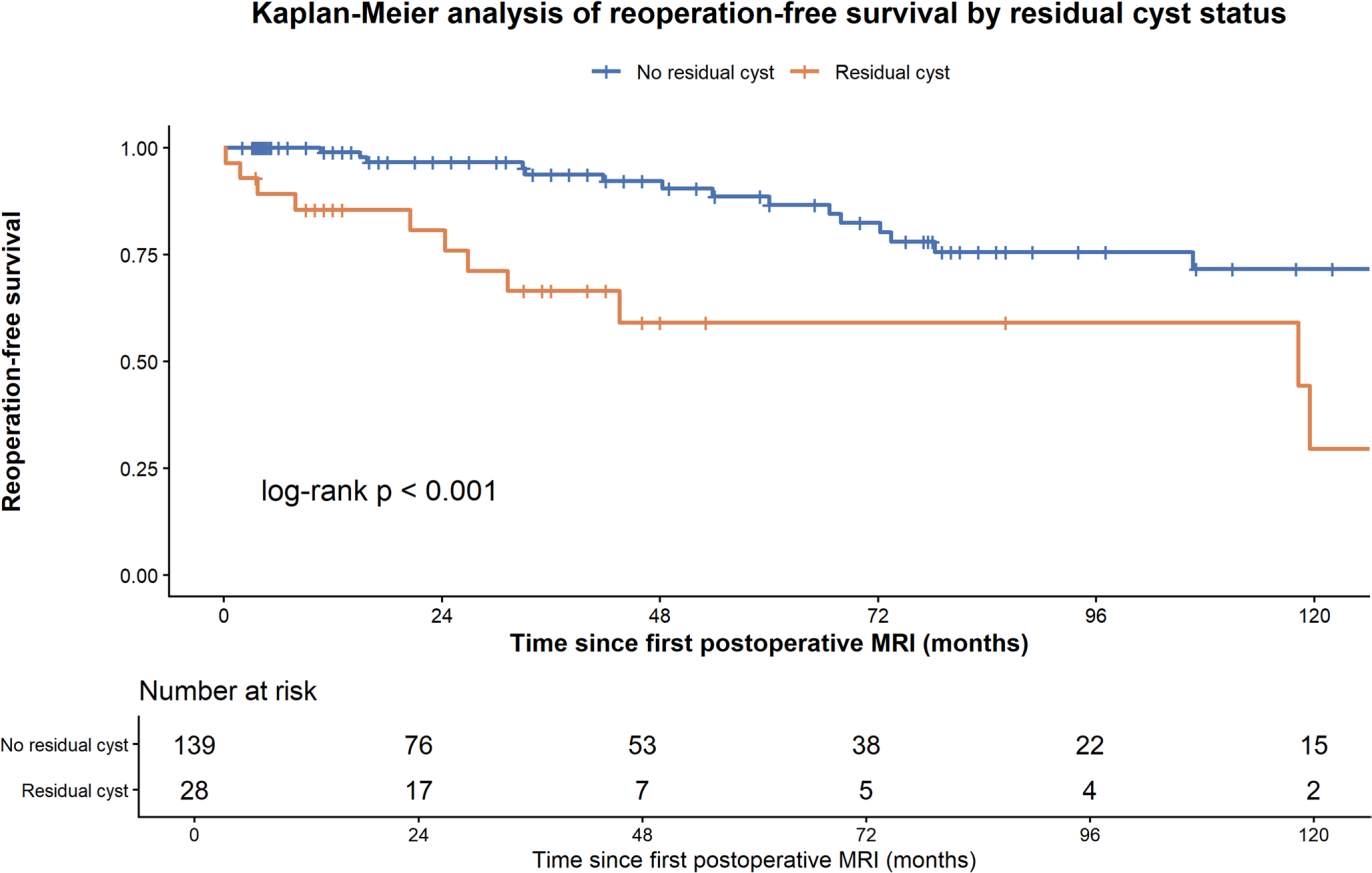
Kaplan-Meier analysis of reoperation-free survival according to residual disease status on first early postoperative MRI. Patients with residual disease showed significantly lower reoperation-free survival than those without residual disease (log-rank *p* < 0.001). Numbers at risk are shown below the plot

In the landmark Kaplan-Meier analysis of these 167 patients, residual cyst on first early postoperative MRI was associated with significantly lower reoperation-free survival than no residual cyst (log-rank *p* < 0.001). Median reoperation-free survival was not reached. Landmark reoperation-free survival rates were 97.1% at 1 year, 89.1% at 3 years, 82.2% at 5 years, and 67.9% at 10 years (Fig. 3).

## Discussion

In this large single-centre cohort of surgically treated RCCs, we integrated volumetric, histopathological and longitudinal endocrine data to refine assessment of pituitary hormonal dysfunction and reoperation risk. Larger cyst volume and inflammatory-metaplastic cyst-wall features were associated with greater pituitary hormonal dysfunction, whereas reoperation was uncommon and typically late. Most clinically relevant net endocrine changes occurred within the first three postoperative months, and AVP-D emerged early and was largely persistent.

The clinical spectrum was consistent with previous surgical series, with headache as the leading manifestation, frequent gonadal dysfunction, fatigue and visual disturbance, and a substantial proportion of incidental findings [2, 4–7]. Although headache in small RCCs is often considered incidental [16, 17], headache was frequent even in modest-volume lesions and was associated with chronic cyst-wall inflammation. Together with the higher frequency of inflammatory wall pathology in symptomatic than incidental lesions, this finding is compatible with an association between cyst-wall inflammation and clinical presentation beyond cyst size alone [3, 6].

Cyst volume was associated with preoperative adenohypophyseal dysfunction, consistent with compressive effects described in prior series [16–18]. In multivariable analyses, larger cyst volume remained independently associated with hyperprolactinaemia and gonadal axis dysfunction, whereas age and sex showed axis-specific associations and cyst location added limited explanatory value. These findings indicate that preoperative pituitary hormonal dysfunction in RCCs is not explained by a single structural parameter, but reflects a multifactorial interplay between lesion size, demographic factors, and anatomical configuration.

Histopathologically, symptomatic RCCs frequently exhibited an inflammatory-metaplastic cyst-wall phenotype associated with greater preoperative pituitary hormonal dysfunction. After FDR correction in univariable analyses, selected inflammatory, metaplastic, and proliferative features were associated with hyperprolactinaemia, gonadal axis dysfunction, SAI, and AVP-D, whereas no significant associations were found for IGF-1-defined somatotropic impairment or thyrotropic dysfunction. These findings are compatible with, but do not prove, a role of local inflammatory and metaplastic processes in endocrine vulnerability [19, 20]. Importantly, none of these features was independently associated with reoperation in exploratory extended Cox models, supporting risk-adapted endocrine follow-up rather than routinely more extensive cyst-wall resection [2, 11, 21].

Preoperative pituitary dysfunction was common and only partially reversible after surgery. Most clinically relevant net endocrine changes occurred within the first three postoperative months: hyperprolactinaemia normalised rapidly, whereas adrenal, thyroid, gonadal, and somatotropic axes showed axis-specific recovery or deterioration. IGF-1-defined somatotropic impairment increased by three months, but interpretation is limited by IGF-1 kinetics, potential non-GH-related influences including BMI increase, and the absence of GH stimulation testing. Thyrotropic findings also require caution, as levothyroxine substitution could not always be reliably attributed to central rather than primary thyroid disease in this retrospective cohort. Beyond three months, no major further net endocrine change was observed in the available paired follow-up cohort, supporting three-month reassessment as a pragmatic clinical milestone. However, long-term findings, including the apparent increase in complete hypopituitarism, should be interpreted cautiously because of attrition and restriction to the paired single-operation cohort.

The postoperative BMI increase observed in paired analyses was not a primary endpoint and should be interpreted cautiously, but may support basic metabolic risk assessment during long-term endocrine follow-up. AVP-D represented a major postoperative morbidity, affecting approximately one quarter of patients and remaining frequently persistent. New-onset AVP-D typically emerged by POD7 with minimal subsequent change, placing our rate at the upper end of the reported 5–20% after RCC surgery [10, 22, 23]. Postoperative AVP-D was unrelated to cyst volume or location; differences by surgical approach were descriptive given the small transcranial subgroup. In contrast, preoperative AVP-D was enriched in cysts with chronic cyst-wall inflammation and higher Ki-67 indices, compatible with an association between inflammatory/proliferative features and neurohypophyseal vulnerability [19]. Given the high incidence of postoperative SIADH, structured peri- and postoperative sodium monitoring remains important [9].

Reoperation occurred in approximately one-sixth of patients after a median of 45.7 months, consistent with published series [4, 7, 24, 25]. Early postoperative residual cyst remained strongly associated with subsequent reoperation in adjusted time-to-event analysis, identifying patients who may require closer radiological surveillance [2, 17, 18, 26]. However, many residuals remained stable, and over half of reoperations occurred after MRI-defined GTR, consistent with later radiological recurrence. These findings underline the need to distinguish radiological residual disease and recurrence from reoperation as a treatment endpoint, and to guide management by growth kinetics, symptoms, and anatomical risk rather than residual status alone [2, 18]. Histopathological features were not independently associated with reoperation in exploratory models, and the higher unadjusted risk in women did not persist after adjustment.

The morbidity profile supports a function-preserving surgical strategy [2, 13, 22, 24, 26]. Serious complications were infrequent, but postoperative water-electrolyte disturbances, including SIADH and AVP-D, remained clinically relevant [7, 9, 24]. Our structured reporting of endocrine, surgical, radiological, and reoperation outcomes aligns with recent core outcome recommendations for pituitary surgery research [27]. Cyst decompression with preservation of pituitary tissue therefore appears appropriate for most patients, as more extensive cyst-wall excision has shown inconsistent benefits for recurrence control while increasing the risk of permanent endocrine deficits [14, 22, 23, 26].

Limitations include the retrospective single-centre design with potential selection/referral bias, restriction to surgically treated patients, and limited quality-of-life and metabolic data beyond BMI. Levothyroxine use could not be reliably attributed to central versus primary thyroid disease; thus, thyrotropic findings represent a pragmatic retrospective endpoint based on low and/or substituted fT4 rather than confirmed central dysfunction. Likewise, low IGF-1 without stimulation testing does not establish GH deficiency; somatotropic results are therefore reported as IGF-1-defined somatotropic impairment. Follow-up was heterogeneous, with paired long-term endocrine data available only in a subset of the single-operation cohort (*n* = 100), introducing potential attrition-related bias and limiting comparison with the full baseline cohort. The small transcranial subgroup (*n* = 10) precluded meaningful inferential comparison of surgical approaches; therefore, all such comparisons should be considered descriptive.

## Conclusion

In this large single-centre cohort of surgically treated RCCs, larger cyst volume and an inflammatory-metaplastic cyst-wall phenotype were associated with greater pituitary hormonal dysfunction at presentation, whereas reoperation was uncommon and typically late. Adenohypophyseal dysfunction was frequent and only partially reversible, with most clinically relevant endocrine changes occurring within the first three postoperative months, supporting standardised reassessment at this time point. Postoperative AVP-D affected approximately one quarter of patients and was largely persistent, underscoring the need for structured water-electrolyte monitoring and long-term endocrine follow-up. Early postoperative MRI residuals were strongly associated with reoperation; however, many residuals remained stable and over half of reoperations occurred after MRI-defined GTR, consistent with late recurrence. These findings support a function-preserving surgical strategy with risk-adapted endocrine and radiological surveillance.

## Supplementary Information

Below is the link to the electronic supplementary material.

Supplementary Material 1

## Data Availability

The data supporting the findings of this study are not publicly available due to patient confidentiality but can be made available upon reasonable request from the corresponding author.
