# Supplementary material for: Long-term endocrine trajectories and reoperation risk after surgery for Rathke’s cleft cysts: a single-center cohort study of 177 patients
Source: Pituitary. 2026 May 18;29(3):89. doi: 10.1007/s11102-026-01698-2 (PMC13183730; doi:10.1007/s11102-026-01698-2)
Supplement: Supplementary file 1 — Supplementary Material 1 [file 11102_2026_1698_MOESM1_ESM.docx]

**Supplementary Material**

**Long-term endocrine trajectories and reoperation risk after surgery for Rathke’s cleft cysts: a single-center cohort study of 177 patients**

**Journal: Pituitary**

**Natalia Kremenevski^1^, Waseem Masalha^1^, Daniel Delev^1^, Roland Coras^2^, Arnd Doerfler^3^, Dieter Henrik Heiland^1^, Oliver Schnell^1^**

**^1^** Department of Neurosurgery, Universitätsklinikum Erlangen, Friedrich-Alexander University Erlangen-Nürnberg, Erlangen, Germany.

**^2^** Department of Neuropathology, Universitätsklinikum Erlangen, Friedrich-Alexander University Erlangen-Nürnberg, Erlangen, Germany.

**^3^** Department of Neuroradiology, Universitätsklinikum Erlangen, Friedrich-Alexander University Erlangen-Nürnberg, Erlangen, Germany.

**Corresponding author:**

**Natalia Kremenevski, MD**

Department of Neurosurgery, Universitätsklinikum Erlangen,

Friedrich-Alexander University Erlangen-Nürnberg,

**Schwabachanlage 6**

**91054 Erlangen, Germany**

**E-mail:** [Natalia.Kremenevski@uk-erlangen.de](mailto:Natalia.Kremenevski@uk-erlangen.de)

**ORCID: 0009-0009-6082-6617**

**Table S1** Axis-specific endocrine changes from preoperative assessment to postoperative day 7 (paired analysis)

| **Axis** | **Pre-op normal (%)** | **POD7 normal (%)** | **New dysfunction (%)** | **Improved (%)** | **McNemar**  **p value** |
| --- | --- | --- | --- | --- | --- |
| Prolactin | 66.7 | 89.8 | 4.2 | 78.0 | <0.001 |
| Corticotropic | 78.0 | 72.9 | 11.6 | 17.9 | 0.093 |
| Gonadal | 59.6 | 52.4 | 16.2 | 6.0 | 0.012 |
| Thyrotropic | 65.5 | 55.4 | 17.2 | 3.3 | <0.001 |
| IGF-1-defined somatotropic | 81.4 | 81.4 | 8.4 | 38.2 | 1.000 |
| AVP-D absent | 91.0 | 75.7 | 17.4 | 6.3 | <0.001 |

**Notes.** Percentages are based on paired datasets for each axis. “Normal” and “abnormal” refer to axis-specific endocrine status at the respective time point. “New dysfunction” denotes the proportion of patients who were normal preoperatively and abnormal at POD7; “Improved” denotes the proportion of patients who were abnormal preoperatively and normal at POD7. For AVP-D, “normal” denotes absence of AVP-D. McNemar’s exact test (two-sided) was used for paired comparisons. Women using oral contraceptives were excluded from gonadal-axis comparisons. For the thyrotropic axis, dysfunction was defined as low/substituted fT4 (low fT4 and/or levothyroxine substitution). For the somatotropic axis, impairment was defined by low age-/sex-adjusted IGF-1 without GH stimulation testing. Abbreviations: AVP-D, arginine vasopressin deficiency; POD7, postoperative day 7.

**Table S2** Axis-specific endocrine changes from postoperative day 7 to 3 months (paired analysis)

| **Axis** | **POD7 normal %** | **3 mo normal %** | **New dysfunction (%)** | **Improved (%)** | **McNemar**  **p value** |
| --- | --- | --- | --- | --- | --- |
| Prolactin | 90.6 | 88.8 | 6.5 | 43.8 | 0.629 |
| Corticotropic | 73.1 | 74.3 | 3.2 | 13.0 | 0.754 |
| Gonadal | 51.6 | 59.4 | 3.8 | 20.0 | 0.008 |
| Thyrotropic | 56.5 | 54.7 | 8.3 | 6.8 | 0.581 |
| IGF-1-defined somatotropic | 81.7 | 74.6 | 10.9 | 9.7 | 0.008 |
| AVP-D absent | 76.0 | 76.0 | 0.8 | 2.4 | 1.000 |

**Notes.** Percentages are based on the paired subset with available 3-month follow-up for each axis; POD7 percentages are recalculated within this paired subset and may therefore differ slightly from overall POD7 values shown elsewhere. “Normal” and “abnormal” refer to axis-specific endocrine status at the respective time point. “New dysfunction” denotes the proportion of patients who were normal at POD7 and abnormal at 3 months; “Improved” denotes the proportion of patients who were abnormal at POD7 and normal at 3 months. For AVP-D, “normal” denotes absence of AVP-D. McNemar’s exact test (two-sided) was used for paired comparisons. Women using oral contraceptives were excluded from gonadal-axis comparisons. For the thyrotropic axis, dysfunction was defined as low/substituted fT4 (low fT4 and/or levothyroxine substitution). For the somatotropic axis, impairment was defined by low age-/sex-adjusted IGF-1 without GH stimulation testing. Abbreviations: AVP-D, arginine vasopressin deficiency; POD7, postoperative day 7.


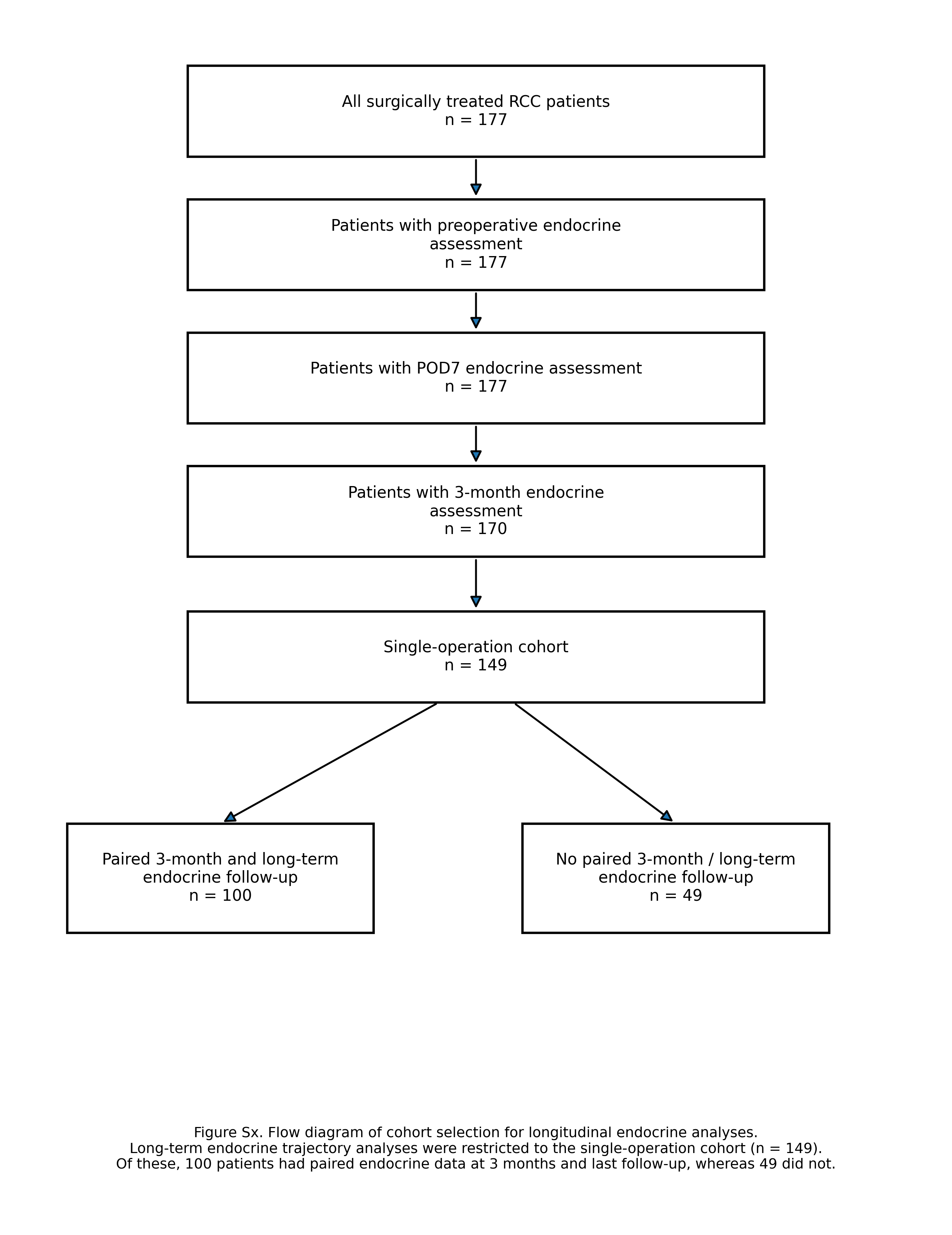


**Fig. S1 Flow diagram of cohort selection for longitudinal endocrine analyses.** Long-term endocrine trajectory analyses were restricted to the single-operation cohort (n=149). Of these, 100 patients had paired endocrine data at 3 months and last follow-up, whereas 49 did not.

**Table S3** Comparison of baseline characteristics between patients with and without paired 3-month/long-term endocrine follow-up within the single-operation cohort

| **Variable** | **No paired 3-month/long-term endocrine follow-up (n=49)** | **Paired 3-month/long-term endocrine follow-up**  **(n=100)** | **p value** |
| --- | --- | --- | --- |
| Age at surgery, years, median (IQR) | 49.3 (32.9-65.5) | 45.4 (28.5-56.8) | 0.131 |
| Cyst volume, cm³, median (IQR) | 0.90 (0.50-1.69) | 0.85 (0.42-1.87) | 0.810 |
| Women, n (%) | 26 (53.1%) | 69 (69.0%) | 0.057 |
| **Cyst location, n (%)** |  |  | 0.158 |
| intrasellar | 12 (24.5%) | 18 (18.0%) |  |
| suprasellar | 4 (8.2%) | 20 (20.0%) |  |
| intra-/suprasellar | 33 (67.3%) | 62 (62.0%) |  |
| **Surgical approach, n (%)** |  |  | 1.000* |
| transsphenoidal | 46 (93.9%) | 93 (93.0%) |  |
| transcranial | 3 (6.1%) | 7 (7.0%) |  |
| **Preoperative pituitary status, n (%)** |  |  | 0.970 |
| normal | 22 (44.9%) | 47 (47.0%) |  |
| partial hypopituitarism | 22 (44.9%) | 43 (43.0%) |  |
| complete hypopituitarism | 5 (10.2%) | 10 (10.0%) |  |

**Notes.** Values are presented as n (%) or median (IQR), as appropriate. Continuous variables were compared using the Mann-Whitney U test. Categorical variables were compared using the χ² test or Fisher’s exact test, as appropriate. *Fisher’s exact test.

**Table S4 Postoperative complications by cyst location and surgical** approach

| **Complication** | **Overall (n=177)** | **Intrasellar (n=32)** | **Suprasellar (n=29)** | **Intra-/suprasellar (n=116)** | **Transsphenoidal (n=167)** | **Transcranial (n=10)** |
| --- | --- | --- | --- | --- | --- | --- |
| SIADH | 41 (23.2%) | 10 (31.3%) | 11 (37.9%) | 20 (17.2%) | 39 (23.4%) | 2 (20.0%) |
| Meningitis | 14 (7.9%) | 0 (0.0%) | 1 (3.4%) | 13 (11.2%) | 14 (8.4%) | 0 (0.0%) |
| CSF leak | 9 (5.1%) | 0 (0.0%) | 1 (3.4%) | 8 (6.9%) | 9 (5.4%) | 0 (0.0%) |
| Transient AVP-D | 7 (4.0%) | 2 (6.3%) | 1 (3.4%) | 4 (3.4%) | 6 (3.6%) | 1 (10.0%) |
| Sinusitis | 6 (3.4%) | 0 (0.0%) | 0 (0.0%) | 6 (5.2%) | 6 (3.6%) | 0 (0.0%) |
| Acute visual deterioration | 3 (1.7%) | 0 (0.0%) | 1 (3.4%) | 2 (1.7%) | 1 (0.6%) | 2 (20.0%) |
| Epistaxis | 2 (1.1%) | 0 (0.0%) | 1 (3.4%) | 1 (0.9%) | 2 (1.2%) | 0 (0.0%) |
| Postoperative haemorrhage | 2 (1.1%) | 0 (0.0%) | 2 (6.9%) | 0 (0.0%) | 0 (0.0%) | 2 (20.0%) |
| Chronic subdural haematoma | 2 (1.1%) | 0 (0.0%) | 1 (3.4%) | 1 (0.9%) | 0 (0.0%) | 2 (20.0%) |
| Sellar abscess | 1 (0.6%) | 0 (0.0%) | 0 (0.0%) | 1 (0.9%) | 1 (0.6%) | 0 (0.0%) |

**Notes.** Values are n (column %). “Transient AVP-D” denotes transient postoperative arginine vasopressin deficiency. The association between complication type and cyst location was not significant (Pearson’s χ²=46.3, df=44, p=0.377). Between-approach statistical comparisons were not performed owing to sparse counts in the transcranial subgroup. Abbreviations: AVP-D, arginine vasopressin deficiency; CSF, cerebrospinal fluid; SIADH, syndrome of inappropriate antidiuretic hormone secretion.
